# Is low-dose computed tomography for lung cancer screening conveniently accessible in China? A spatial analysis based on cross-sectional survey

**DOI:** 10.1186/s12885-024-12100-4

**Published:** 2024-03-14

**Authors:** Jay Pan, Jianjian Wang, Wenjuan Tao, Chaohui Wang, Xiaojun Lin, Xiuli Wang, Ruicen Li

**Affiliations:** 1 HEOA Group, West China School of Public Health and West China Fourth Hospital, Sichuan University, Chengdu, China; 2 Institute for Healthy Cities and West China Research Center for Rural Health Development, Sichuan University, Chengdu, China; 3 Institute of Hospital Management, West China Hospital, Sichuan University, Chengdu, China; 4 Health Management Center and General Practice Medical Center, West China Hospital, Sichuan University, Chengdu, China

**Keywords:** Lung cancer screening, Low-dose computed tomography, Spatial analysis, Health disparities

## Abstract

**Background:**

Regular Low-Dose Computed Tomography (LDCT) for lung cancer high-risk population has been proved to improve health outcomes and relieve disease burden efficiently for both individual and society. With geographical impedance becoming the major barrier preventing patients from getting timely healthcare service, this study incorporated health seeking behavior in estimating spatial accessibility of relative scarce LDCT resource in China, thus to provide real-world evidence for future government investment and policy making.

**Methods:**

Taking Sichuan Province in southwest China as the study area, a cross-sectional survey was first carried out to collect actual practice and preferences for seeking LDCT services. Using Computed Tomography (CT) registration data reported by owner institutions representing LDCT services capacity, and grided town-level high-risk population as demand, the Nearest Neighbor Method was then utilized to calculate spatial accessibility of LDCT services.

**Results:**

A total of 2,529 valid questionnaires were collected, with only 34.72% of the high-risk populations (746 individuals) followed the recommended annual screening. Participants preferred to travel to municipal-level and above institutions within 60 min for LDCT services. Currently, every thousand high-risk populations own 0.0845 CT scanners in Sichuan Province, with 96.95% able to access LDCT within 60 min and over half within 15 min. Urban areas generally showed better accessibility than rural areas, and the more developed eastern regions were better than the western regions with ethnic minority clusters.

**Conclusions:**

Spatial access to LDCT services is generally convenient in Sichuan Province, but disparity exists between different regions and population groups. Improving LDCT capacity in county-level hospitals as well as promoting health education and policy guidance to the public can optimize efficiency of existing CT resources. Implementing mobile CT services and improving rural public transportation may alleviate emerging disparities in accessing early lung cancer detection.

**Supplementary Information:**

The online version contains supplementary material available at 10.1186/s12885-024-12100-4.

## Background

Lung cancer is the most prevalent and deadly malignancy worldwide [1]. Research indicates that global cancer costs will reach $25.2 trillion over the next 30 years, with lung cancers, tracheal, and bronchus comprising the largest proportion at 15.4% ($3.9 trillion). China will bear the largest economic costs of cancers in absolute terms, accounting for 24.1% of the total global burden [2]. According to the latest data released by the National Cancer Center (NCC) in 2016, China had an incidence rate of 59.89 per 100,000 for lung cancer, with the mortality rate being 47.51% [3].

The timing of clinical diagnosis greatly impacts the health outcomes of lung cancer patients, with lower 5-year survival rates associated with later stages. Stage I patients exhibit a 5-year survival rate of 55.5%, while the rate for stage IV patients is only 5.3% [4]. However, the majority of lung cancer cases in China are diagnosed at advanced stages (III-IV), constituting 64.6% of the total cases [4]. With broad agreement reached on that early screening for high-risk population is of paramount importance in the management of lung cancer, more attention has been paid to the development of screening technique and nationwide implementation.

There is compelling evidence that Low-Dose Computed Tomography (LDCT) lung cancer screening significantly reduces mortality rates in patients [5–8]. Many countries and organizations recommend LDCT as the primary method for early lung cancer screening and matched policy has been proposed [9–11]. In March 2021, the United States Preventive Services Task Force (USPSTF) updated their lung cancer screening guidelines [12]. According to the latest recommendations, 14.5 million Americans will be eligible for screening, which is an increase of 6.5 million individuals compared with the previous guidelines [13, 14]. However, even in this context, less than 5% of the eligible population has undergone LDCT screening as required [15]. The geographic location of institutions providing service and public compliance have been identified as major influencing factors [8].

With increasing lung cancer disease burden, the central government of China has incorporated lung cancer screening in several nationwide public health projects. The more prominent ones are the National Major Public Health (NMPH) program in 2005 and the Urban Cancer Early Detection and Early Diagnosis (UCEDED) program in 2012 [16]. These initiatives have demonstrated gradual improvements in compliance rates for lung cancer screening in China [17]. However, the LDCT screening rate in most cities remains below 50% [18–21], with significantly lower participation rates in rural areas, while rural areas are often where lung cancer vulnerable groups are more concentrated. To efficiently alleviate lung cancer disease burden as well as improve health equity as proposed in the national “Healthy China” strategy, promoting LDCT coverage and reducing urban-rural disparities is crucial. In China, healthcare resources are unevenly distributed with gathering centers clustered in developed areas [22–25]. Being one of the typical high-value healthcare equipment, Computed Tomography (CT) scanners which are essential for LDCT are usually owned by large-scale hospitals that have sufficient need and technicians for CT services. The inadequate and uneven availability of CT scanners has been proved to significantly impact the accessibility of LDCT services and lung cancer screening in China, especially in rural areas of the western region [23, 26].

The convenience of overcoming geographical barriers and receiving healthcare services is known as spatial accessibility and is gaining increasing attention by researchers and policy makers [27]. Based on annual data from China’s rural poverty monitoring reports, geographical barriers have surpassed economic burdens as the primary obstacle hindering timely access to healthcare services [28, 29]. Apart from allocation of healthcare resources which was being widely studied [30, 31], healthcare seeking behavior from the demand side is also essential for accurate assessment of spatial accessibility, which includes the preference of healthcare institutions, travel mode, as well as travel impendence. Given the growing burden of lung cancer, and urgent requirement for extension of LDCT in China, a pilot study was carried out in the fifth-largest Sichuan Province. Actual practice and preference for LDCT were investigated through questionnaire. Then, the results were integrated into the Nearest Neighbor Method to assess the rationality of LDCT resource allocation. Based on this, political guidance was proposed to further promote the coverage of lung cancer early screening and diagnosis.

## Methods

### Design

The study was carried out in two phases. Firstly, a cross-sectional survey was conducted to collect basic information on lung cancer screening. Secondly, spatial accessibility to LDCT was calculated through defining accessibility, data collection, and statistical analysis.

### Phase 1: cross-sectional survey

Survey participants were selected through Stratified Proportional Sampling (SPS) in combination with convenience sampling and snowball sampling. The survey was conducted using a web-based platform (the questionnaires were completed by Survey Star via WeChat) between August 27, 2022, and September 9, 2022, in Sichuan, China.

#### Study area

Sichuan Province is located in southwestern China and comprises 21 municipalities and prefectures. According to the 7th national population census, the total population is 83.71 million, with 56.73% being urban population. Because of high smoking rate and traditional lifestyle, lung cancer is the highest incidence rate cancer in Sichuan Province [32]. As the fifth-largest province both in area and population in China, Sichuan can be divided into two distinctly different areas similar to the whole China, which are the eastern region, characterized by flat terrain, higher economic development, and dense population distribution (represented by Chengdu city), and the western region, characterized by vast land, sparse population, lower economic development, and a concentration of ethnic minorities (primarily in Ganzi Tibetan Autonomous Prefecture, Aba Tibetan and Qiang Autonomous Prefecture, and Liangshan Yi Autonomous Prefectural). Sichuan exhibits a miniature of China and research from Sichuan has wide applicability.

#### Survey participants

The inclusion criteria for survey participants consists of four aspects. (1) Residency: Individuals who have lived in Sichuan Province for at least one year. (2) Age: The general population between the ages of 40 and 74 who are asymptomatic for lung cancer and have no lung cancer history. (3) Cognitive ability: Individuals must have the cognitive capacity to participate in the survey. (4) Willingness: Participation is voluntary based on informed consent, including the right to refuse or withdraw without any disadvantages.

#### Survey information

The design of the questionnaire is described in detail in another series study, and data utilized in this research includes four aspects. (1) Basic characteristics of the survey participants. (2) Risk factors for lung cancer, including smoking history, smoking cessation, passive smoking, history of chronic respiratory diseases, occupational exposure history, and family history of lung cancer. (3) Lung cancer screening information, such as whether participants have undergone CT lung cancer screening, the methods used for screening, and the screening institutions. (4) Preference for seeking lung cancer screening services, including preferred mode of transportation and time of travel.

### Phase 2: calculating spatial accessibility

#### Data collection

Three types of data are essential in calculating spatial accessibility, including supply-side data, demand-side data, and spatial cost data as listed in Table 1. In this study, the spatial distribution of CT scanners was utilized as LDCT service capacity, while LDCT doctors were not included due to two main reasons. Firstly, as one of the representative high-value medical equipment, the purchase of CT scanners is governed by the government, and capability of healthcare institutions to operate CT scanners (including healthcare professionals operating CT scanners which were categorized as part of image technicians) is essential. Secondly, implementation of a cloud film system designed for instant storage and retrieval of images, facilitating multimedia remote access, and enabling remote diagnostics is quite common in China with rapid development of information technologies. Although doctors making diagnose based on CT scan results is rare in China, CT scan results can be transferred to doctors in high level hospitals to make diagnose and return results in short time.

**Table 1:** Data description and source for spatial accessibility calculation

Data type	Data description	Data source
Supply data	Spatial distribution of CT scanners, including CT model, purchase date, quantity purchased in the same batch, and owner institution information including name, type, and address.	The equipment registration platform of the Sichuan Provincial Health Commission’s Information Center.
Demand data	Spatial distribution of high-risk population for lung cancer. For more accurate assessment, township level aggerated high-risk population was further allocated to 1 km*1km population grids.	Age and gender-specific population data at the township level from the Sichuan Provincial Health Commission.
		Population density at 1 km*1km grid scale from WorldPop.
Spatial cost data	Travel time along the road network from demand location to institution providing LDCT was utilized as spatial cost.	National transportation network data at a 1:100 scale published by the National Geomatics Center of China.

#### Data pretreatment

Under ArcGIS environment, healthcare institutions owing CT scanners were saved as points with number of CT scanners as service capacity. The spatial distribution of high-risk population for lung cancer was saved as 1 km*1km population grids following three steps. (1) Combing the criteria for identifying high-risk population in the “China Lung Cancer Screening and Early Detection and Treatment Guidelines (2021, Beijing)” and the “Consensus on Lung Cancer Screening and Management in China” with information from the cross-sectional surveys, a high-risk ratio was calculated. (2) The ratio was then combined with age and gender-specific population data to get the number of high-risk populations in every township in Sichuan. (3) WorldPop population density data was used as weight to downscale township level high-risk population to every 1 km*1km population grid [33]. Following travel habit in China, three transportation networks were constructed in ArcGIS, which were walking (with an average speed of 5 km/h), bicycle/electric vehicle travel (with an average speed of 15 km/h), and driving (with an average speed ranging from 5 km/h to 75 km/h). The data were then integrated into basic administrative boundaries to establish a geographical information database.

#### Statistical analysis

The Nearest Neighbor Method was used to analyze spatial access to LDCT services, with the primary indicator being the time it takes for patients at every resident grid to reach the nearest healthcare institution that provides LDCT services. A comprehensive comparison between different regions, urban and rural areas was conducted to further enhance the analysis.

In cooperation with the survey, healthcare institutions were classified into four levels (provincial-level and above, municipal-level, county-level, and others). Three travel modes were taken into account, and the shortest travel time under different travel mode to different healthcare institutions were calculated separately. For each resident grid, the shortest travel time was classified into 0–15 min, 15–30 min, 30–60 min, 60–120 min, and over 120 min for better understanding and comparison.

## Results

### Phase 1: cross-sectional survey

The cross-sectional survey yielded a total of 2,529 valid questionnaires from 21 municipals of Sichuan Province, with 746 (29.50%) being high-risk. The proportion of respondents collected from every municipal/prefecture was generally consistent with provincial demographics as listed in Table S1. The average age of respondents was 49.57 ± 7.78 years, with females making up over half of the sample (57.49%). The majority of respondents resided in urban areas (79.04%), and 47.93% had attained a university education and above. Overall, 61.53% (1,556 individuals) have participated in lung cancer screening, with the high-risk group accounting for 29.11% (453 individuals) of this number. However, only 34.72% (259 individuals) of the high-risk population adhered to the annual screening recommendation. Municipal hospitals were the primary choice for most participants (1,007 individuals accounting for 64.72%), followed by provincial hospitals (347 individuals accounting for 22.30%). No significant differences were observed in screening status or choice of institution between non-high-risk and high-risk groups. A comprehensive overview of participant characteristics and the practical aspects of lung cancer screening is documented in Table 2.

**Table 2:** Characteristics and actual practice of lung cancer screening among survey participants

Items	Classification	Overall population *N* = 2529(%)	Non-high-risk population *N* = 1783(%)	High-risk population *N* = 746(%)	p value†
Sex					0.383
	Female	1454(57.49)	1035(58.05)	419(56.17)	
	Male	1075(42.51)	748(41.95)	327(43.83)	
Age, years					0.000
	40 ~ 49	1328(52.51)	1028(57.66)	300(40.21)	
	50 ~ 59	924(36.54)	587(32.92)	337(45.17)	
	60 ~ 69	240(9.49)	142(7.96)	98(13.14)	
	70 ~ 74	37(1.46)	26(1.46)	11(1.47)	
Location					0.074
	Urban	1999(79.04)	1426(79.98)	573(76.81)	
	Rural	530(20.96)	357(20.02)	173(23.19)	
Education level					0.000
	Primary school or below	132(5.22)	83(4.66)	49(6.57)	
	Junior middle school	277(10.95)	175(9.81)	102(13.67)	
	High school	338(13.36)	213(11.95)	125(16.76)	
	Vocational diploma	570(22.54)	393(22.04)	177(23.73)	
	University or above	1212(47.93)	919(51.54)	293(39.27)	
Monthly income (CNY)					0.712
	≤ 3000	335(13.25)	231(12.96)	104(13.94)	
	3000–5000	490(19.38)	350(19.63)	140(18.77)	
	5000–8000	800(31.63)	556(31.18)	244(32.71)	
	≥ 8000	904(35.75)	646(36.23)	258(34.58)	
Screening status					0.065
	Never	812(32.11)	573(32.14)	239(32.04)	
	Has done	741(29.30)	547(30.68)	194(26.01)	-
	Once a year	815(32.23)	556(31.18)	259(34.72)	-
	Unclear	161(6.37)	107(6.00)	54(7.24)	-
Screening institutions	Total	1556(61.53)	1103(61.86)	453(60.72)	0.017
	Township health centers/Community health centers	12(0.77)	8(0.73)	4(0.88)	
	District/County-level hospitals	190(12.21)	127(11.51)	63(13.91)	-
	Municipal-level hospitals	1007(64.72)	743(67.36)	264(58.28)	-
	Provincial hospitals	347(22.30)	225(20.40)	122(26.93)	-

†Chi-squared test with a significance level of *p* ≤ 0.05

Preference for travel mode and acceptable travel time to different levels of healthcare institutions is shown in Table 3. In general, more than 90% of participants preferred municipal and above level healthcare institutions for screening service. The majority of participants (1,841 individuals accounting for 72.80%) opted to drive/take public transportation, and the acceptable travel time is mostly within 30 min. For participants who rely on walking or using bicycle/electric vehicles, the proportion that can accept travel time exceeding 30 min is relatively low (3.08%, 78 individuals). However, for residents who rely on driving/taking public transportation, a higher proportion can accept longer travel time, with 12.89% (326 individuals) able to accept travel time exceeding 60 min. As the level of healthcare institutions decreases from provincial to municipal and county level, the proportion of participants relying on public transportation and their acceptable travel time decreases subsequently.

**Table 3:** Acceptable travel time to healthcare institutions under different travel modes

Type of healthcare institutions		Travel mode		Travel Time (min)			
Classification	Number (%)	Classification	Number (%)	0–15	15–30	30–60	> 60
Healthcare institutions above provincial-level	869 (34.36%)	Walking	69(7.94%)	40(57.97%)	25(36.23%)	4(5.80%)	0(0.00%)
		Bicycle/Electric vehicle	45(5.18%)	14(31.11%)	23(51.11%)	7(15.56%)	1(2.22%)
		Driving/Public transportation	755(86.88%)	39(5.17%)	214(28.34%)	264(34.97%)	238(31.52%)
Municipal-level healthcare institutions	1419 (56.11%)	Walking	286(20.16%)	124(43.36%)	117(40.91%)	42(14.69%)	3(1.05%)
		Bicycle/Electric vehicle	151(10.64%)	54(35.76%)	87(57.62%)	9(5.96%)	1(0.66%)
		Driving/Public transportation	982(69.20%)	201(20.47%)	495(50.41%)	213(21.69%)	73(7.43%)
County-level healthcare institutions	203 (8.03%)	Walking	74(36.45%)	23(31.08%)	45(60.81%)	5(6.76%)	1(1.35%)
		Bicycle/Electric vehicle	41(20.20%)	16(39.02%)	21(51.22%)	3(7.32%)	1(2.44%)
		Driving/Public transportation	88(43.35%)	17(19.32%)	36(40.91%)	22(25.00%)	13(14.77%)
Other healthcare institutions	38 (1.50%)	Walking	15(39.47%)	12(80.00%)	3(20.00%)	0(0.00%)	0(0.00%)
		Bicycle/Electric vehicle	7(18.42%)	2(28.57%)	4(57.14%)	1(14.29%)	0(0.00%)
		Driving/Public transportation	16(42.11%)	3(18.75%)	6(37.50%)	5(31.25%)	2(12.50%)

### Phase 2: spatial accessibility

#### Overall accessibility of LDCT services

**Fig. 1:**
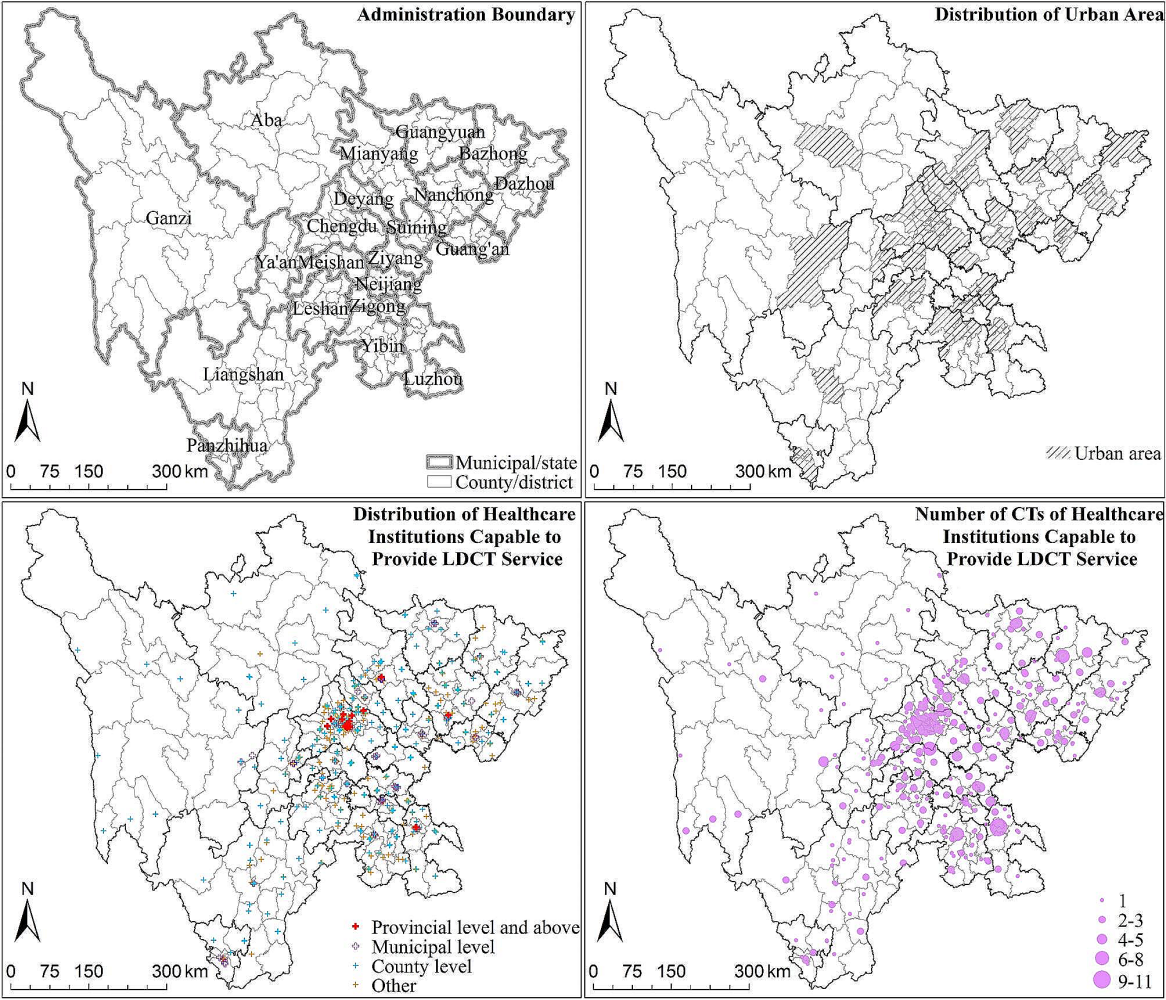
Spatial distribution of CT scanners configuration in Sichuan Province

In 2021, Sichuan Province had a total of 1,075 CT scanners distributed in 706 healthcare institutions available for LDCT services, with an average of 0.0845 CT scanners per thousand high-risk populations. The 23 healthcare institutions above provincial-level own 65 CT scanners, providing 6.05% of CT accessibility (0.0051). The 66 municipal-level healthcare institutions own 181 CT scanners, providing 16.84% of CT accessibility (0.0142). The 252 county-level healthcare institutions own 416 CT scanners, providing the highest percentage of 38.70% of the total CT accessibility (0.0327). And 365 other types of healthcare institutions own 413 CT scanners, providing 38.42% of CT accessibility (0.0324) (Fig. 1).

From an urban-rural perspective, slightly more high-risk populations live in urban areas (607) compare with rural areas (139), and the number of CT scanners in urban areas (710) is approximately twice that of rural areas (365). The density of CT scanner is 0.1109 per thousand high-risk populations in urban areas and 0.0578 in rural areas. Additionally, higher-level healthcare institutions (municipal and above level) are generally concentrated in urban areas.

#### Minimum travel time to access LDCT services

The percentage of high-risk population and area within different travel zones to LDCT healthcare institutions is listed in Table 4. Under the mode of driving/taking public transportation, 99.52% of high-risk populations can reach the nearest healthcare institution for LDCT services within 120 min, of which 96.95% can reach within 60 min and 58.83% within 15 min. In the case of bicycle/electric vehicle travel, 16.87% of high-risk populations need more than 120 min and 23.43% can reach the nearest healthcare institution within 15 min. Under the walking mode, only 28.51% of high-risk populations can access LDCT services within 60 min, and more than half need over 120 min. For area coverage, the peaks generally fall within larger travel zones compared to population coverage.

**Table 4:** Minimum travel time to obtain LDCT services

Minimum Travel Time (min)	0–15	15–30	30–60	60–120	> 120
Proportion of high-risk population					
Driving/Public transportation	58.83%	25.09%	13.04%	2.57%	0.48%
Bicycle/Electric vehicle	23.43%	13.20%	19.95%	26.55%	16.87%
Walking	6.90%	9.66%	11.96%	15.04%	56.44%
Area proportion					
Driving/Public transportation	13.18%	18.57%	24.28%	23.69%	20.28%
Bicycle/Electric vehicle	1.22%	2.74%	7.81%	15.80%	72.43%
Walking	0.18%	0.44%	1.39%	3.97%	94.02%

Considering different levels of healthcare institutions, county-level healthcare institutions and other healthcare institutions make predominant contributions to ensuring LDCT coverage (Table 5). Under driving/taking public travel mode, the general proportion of high-risk population covered within 15 min is 58.83%, meanwhile county-level healthcare institutions and other healthcare institutions covered 49.97% and 49.07% of high-risk population respectively, and healthcare institutions above provincial-level, municipal-level healthcare institutions covered only 13.76% and 23.26% of high-risk population. Similar trends are observed in the other two travel modes, and with travel speed decreases, the contribution of county-level healthcare institutions and other healthcare institutions on the general coverage decreases, also the predominant role transferred from county-level healthcare institutions to other healthcare institutions. The less overlap between healthcare institutions’ coverage under slower transportation and the larger number of other healthcare institutions which are more clustered in spatial distribution should be the main reason. In terms of area coverage, the significance of county-level healthcare institutions and other healthcare institutions in ensuring LDCT coverage becomes more evident (see Table S2).

**Table 5:** Population distribution of minimum travel time to obtain LDCT services in different healthcare institutions

Minimum Travel Time (min)	0–15	15–30	30–60	60–120	> 120
Driving/Public transportation					
Proportion of total coverage high-risk population	58.83%	25.09%	13.04%	2.57%	0.48%
Proportion of healthcare institutions above provincial-level	13.76%	9.52%	27.32%	34.07%	15.33%
Proportion of municipal-level healthcare institutions	23.26%	25.13%	35.09%	13.45%	3.07%
Proportion of county-level healthcare institutions	49.97%	28.88%	17.21%	3.43%	0.51%
Proportion of other healthcare institutions	49.07%	25.93%	18.91%	4.52%	1.57%
Bicycle/Electric vehicle					
Proportion of total coverage high-risk population	23.43%	13.20%	19.95%	26.55%	16.87%
Proportion of healthcare institutions above provincial-level	3.40%	3.48%	5.74%	7.40%	79.98%
Proportion of municipal-level healthcare institutions	7.08%	6.09%	7.74%	17.18%	61.91%
Proportion of county-level healthcare institutions	13.23%	13.35%	20.40%	28.64%	24.38%
Proportion of other healthcare institutions	17.68%	11.85%	17.17%	24.49%	28.81%
Walking					
Proportion of total coverage high-risk population	6.90%	9.66%	11.96%	15.04%	56.44%
Proportion of healthcare institutions above provincial-level	0.71%	1.33%	2.60%	4.45%	90.92%
Proportion of municipal-level healthcare institutions	1.31%	2.89%	5.34%	6.17%	84.29%
Proportion of county-level healthcare institutions	2.24%	5.40%	10.35%	15.62%	66.38%
Proportion of other healthcare institutions	4.59%	7.33%	10.21%	13.53%	64.35%

In Sichuan Province, urban areas account for 17.87% of the total area but cover 50.36% of the population. Higher-level healthcare institutions, such as provincial-level and municipal-level, are predominantly concentrated in urban areas. This concentration results in higher accessibility and quality of LDCT services in urban areas compared to rural areas. The disparity in terms of area coverage is greater than the disparity in population coverage, with the greatest difference observed in walking mode and the smallest in driving/taking public transportation (see Table S3-S5). Under driving/taking public travel mode, 73.71% of the urban population can reach the nearest healthcare institution for LDCT services within 15 min, while the rate is only 43.86% in rural areas (see Table S3). In terms of area coverage, 32.38% of the urban area falls within the 15-minute service range of healthcare institutions, but only 9.09% in rural areas (see Table S3). Moreover, the proportion of the high-risk population covered within the 15-minute range of healthcare institutions above provincial-level and municipal-level healthcare institutions in urban areas is 26.70% and 43.41%, with only 0.67% and 2.87% in rural areas in driving/taking public transportation, the disparity reaches nearly 40 and 15 times respectively (see Table S4). In terms of area, the ratios are 100 and 28 respectively (see Table S5).

**Fig. 2:**
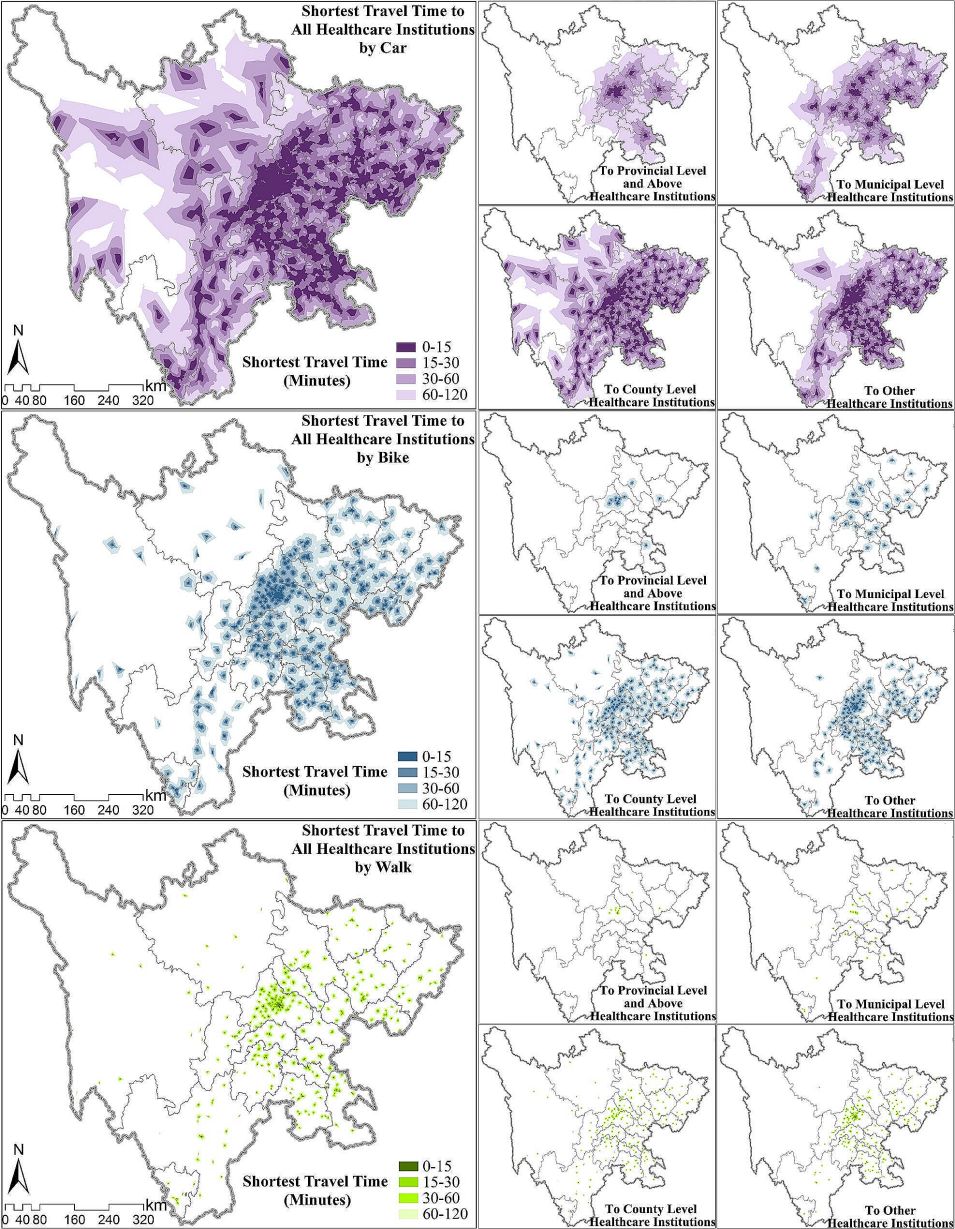
Minimum travel time for high-risk population to obtain LDCT services under different travel modes

From the spatial perspective, residents facing challenges in accessing LDCT services timely are primarily concentrated in the western mountainous areas of Sichuan Province, with Ganzi Tibetan Autonomous Prefecture, Liangshan Yi Autonomous Prefectural, and Aba Tibetan and Qiang Autonomous Prefecture being typical. The average travel time in eastern region is significantly shorter than that in the western region, with the best accessibility observed in Chengdu as it is the provincial capital (Fig. 2, Table S6). In Chengdu 89.74% of the population can reach the nearest healthcare institution for LDCT services within 15 min, followed by Panzhihua (78.38%) and Deyang (76.88%). Oppositely, in Ganzi Tibetan Autonomous Prefecture, 27.48% of the high-risk populations are more than 120 min away from the nearest healthcare institutions capable of providing LDCT services, while the rate in Aba Tibetan and Qiang Autonomous Prefecture and Liangshan Yi Autonomous Prefectural are 7.63% and 3.10% respectively. Apart from the three ethnic minority autonomous prefectures, almost all population can access LDCT services within 120 min. Considering different levels of healthcare institutions, almost 100% of the population can access all four levels of healthcare institutions within 60 min in Chengdu under the driving/taking public travel mode. Meanwhile, in Bazhong, Ganzi Tibetan Autonomous Prefecture, Liangshan Yi Autonomous Prefectural, and Panzhihua, there are no access to healthcare institutions above provincial-level. Under the driving/taking public travel mode, only county-level healthcare institutions can cover over 90% of the population within 60 min, except for Ganzi Tibetan Autonomous Prefecture, Aba Tibetan and Qiang Autonomous Prefecture, and Liangshan Yi Autonomous Prefectural (see Table S7).

## Discussion

Actual practices and preferences for LDCT lung cancer screening in Sichuan Province were studied through a province-wide cross-sectional survey. The results were integrated into spatial accessibility models to reveal the current rationality of CT resource allocation. The findings indicate that further efforts are needed to enhance awareness and compliance with early and regular screening among the high-risk population for lung cancer. The spatial coverage of LDCT services in Sichuan Province is generally satisfactory, but more effort is required to improve equity, especially in the less developed rural western regions.

The current spatial distribution of LDCT resources in Sichuan Province is relatively reasonable, which provides favorable bases for future nationwide lung cancer management strategies. Under the strategic framework of the national “Healthy China” action plan, the National Cancer Center actively implements cancer prevention and control strategies, including promoting screening and early diagnosis and treatment of malignant tumors such as lung cancer. With increasing recognition of LDCT for lung cancer screening, the availability of sufficient and convenient CT scanners is essential for the widespread implementation of early lung cancer screening [34]. This research shows that more than 95% of the high-risk populations can access LDCT within 60 min through driving/taking public transportation, and 16.56% can walk to the nearest LDCT institution within 30 min, representing generally convenient access to LDCT services.

However, equity in accessing LDCT services remains a major concern for policymakers in Sichuan Province. Equal and accessible comprise the two main goals of healthcare resource allocation in China’s recent government reports, but disparities between rural and urban areas, as well as the more developed eastern and less developed western regions of Sichuan Province, are significant. From the quantity aspect, travel time is generally shorter in better-developed urban areas in the eastern region. From the quality aspect, the difference of spatial access to higher-level healthcare institutions is larger than lower-level healthcare institutions. Moreover, the disparity between urban and rural areas is closely related to the degree of transportation limitations, with greater differences in LDCT services accessibility between urban and rural areas in regions with more restricted transportation options.

Conflict exists between LDCT resource allocation among different levels of healthcare institutions and public preference. Our findings reveal that in Sichuan Province, high-level healthcare institutions at the municipal and provincial levels provide only 22.89% CT accessibility, which is often concentrated in urban areas. In contrast, county-level medical institutions offer 38.70% CT accessibility. Although county-level healthcare institutions and other healthcare institutions (mostly private hospitals) make significant contributions to ensuring LDCT coverage, more than 90% of participants preferred municipal and above level healthcare institutions for screening service. The actual 87.02% of respondents accepting LDCT services at municipal and above level healthcare institutions confirmed the selection biases.

In addition, adherence to annual lung cancer screening among high-risk populations is suboptimal. In Sichuan, lung cancer has the highest incidence rate among all malignancies. However, survey data reveals that only 34.72% of those at high risk are undergoing the recommended yearly screenings, a trend consistent with results from other regions in China [18–21]. Although this rate exceeds that of the United States in 2017 (14.4%, 6.5–18.1%) [34], the economic benefits of LDCT screening need to be based on a participation rate as high as 95% according to research from the National Lung Screening Trial (NLST) in the US [6].

It is important to consider the undiscovered lung cancer burden in LDCT resource allocation in China. Recent research revealed a rising incidence of lung cancer in rural Sichuan, which is resulted from improved healthcare access and early detection initiatives in rural settings. Meanwhile, in urban areas, incidence of lung cancer is experiencing a decline due to heightened awareness about lung cancer risks in cities [35]. Studies show that that individuals who undergo physical examinations, have a family history of lung cancer, or receive chest X-rays or LDCT are more knowledgeable about lung cancer and aware that LDCT can effectively detects early-stage lung cancer [36]. With social development, the presented incidence of lung cancer in rural area will keep rising, while demand for LDCT screening in both rural and urban area will increase.

Meanwhile, challenges faced by ethnic minorities should be emphasized. Even in sparse populated ethnic minority regions, ethnic minorities are mostly distributed in vast rural area with underdeveloped public infrastructures. Because of the sparse population distribution and lower economic level, healthcare institutions in ethnic minority areas are usually government owned, comparatively lower in level, and fewer in number. Coupled with insufficient health awareness among ethnic minority residents, incidence of lung cancer among ethnic minority groups is a great challenge. Special attention should be paid to improving LDCT coverage in ethnic minority regions.

In summary, regional inequality in lung cancer screening is an important factor contributing to persistent disparities in lung cancer incidence and mortality rates. More efforts should be devoted to correctly guiding the public to both improve accessibility and enhance resource efficiency. Recommendations for the government aimed at improving LDCT screening and thus relieving the lung cancer disease burden were proposed as follows. Firstly, service capacity of LDCT screening in grassroot healthcare institutions should be improved by strengthening topical training and standardized management, meanwhile, health education to the public should be promoted widely to improve compliance among high-risk patients and enhance trust in lower-level healthcare institutions, to promote efficiency of CT resources in county level hospital represented low level healthcare institutions. Secondly, new techniques such as mobile CT vehicle and regular government leaded community-based lung cancer screening activities should be carried out especially in sparsely populated resource-limited rural areas and ethnic minority areas to address the issue of uneven spatial allocation of CT scanners [26]. Thirdly, efforts should be made to improve public infrastructure, such as public beneficial “rural buses” in remote areas to enhance mobility and facilitating access to LDCT services.

This study is the first exploration of spatial accessibility of lung cancer screening services in China. We utilized CT scanner information from government databases and conducted a representative cross-sectional survey to gain insights into residents’ preferences and willingness to access LDCT services. The innovative use of Nearest Neighbor Method provided valuable information on the spatial accessibility of LDCT services. However, our study has several limitations. Firstly, Sichuan Province was taken as a representative of China, but comparison with other regions within and outside of China was not included due to data limitation. Secondly, we assumed that participants in the cross-sectional survey have equal opportunities to access LDCT services, without considering potential disparities among different socioeconomic groups, non-spatial factors such as service quality, insurance types, and reimbursement schemes that may also affect spatial accessibility and should be considered in future research [25]. Thirdly, the service capacity of CT scanners as well as availability of LDCT doctors was not considered in this study due to data limitation.

## Conclusions

In conclusion, the spatial coverage of CT scanners in Sichuan Province is relatively good, but still need improvement in terms of equity and efficiency. To promote LDCT services for early diagnosis and treatment of lung cancer, it is important to enhance the capacity of county-level healthcare institutions, promote health education to the public, and innovate new techniques such as mobile CT. Under policy of prevention first and integrating prevention with control, it is essential to conduct comparable studies across different regions to understand diverse health needs, thus provide real-world evidence for future resource allocation and policy making. The proposed approach is recommended to implement nationwide to facilitate the optimization of health resource allocation and ensure equitable access to healthcare services.

### Electronic supplementary material

Below is the link to the electronic supplementary material.

Supplementary Material 1

## Data Availability

The datasets used and analysed during the current study are available from the corresponding author on reasonable request.

## Abbreviations

LDCT: Low-dose computed tomography
CT: Computed tomography
