# Supplementary material for: Is low-dose computed tomography for lung cancer screening conveniently accessible in China? A spatial analysis based on cross-sectional survey
Source: BMC Cancer. 2024 Mar 14;24:342. doi: 10.1186/s12885-024-12100-4 (PMC10941474; doi:10.1186/s12885-024-12100-4)
Supplement: Supplementary file 1 — Supplementary Material 1 [file 12885_2024_12100_MOESM1_ESM.docx]

**Table S1** Distribution of the number of respondents in each city of Sichuan Province

| Cities (Prefectures) | Population (million) | Population proportion | Minimum sample size | Number of questionnaires |
| --- | --- | --- | --- | --- |
| Chengdu | 20.938 | 0.2502 | 71 | 664 |
| Zigong | 2.489 | 0.0297 | 8 | 51 |
| Panzhihua | 1.212 | 0.0145 | 4 | 25 |
| Luzhou | 4.254 | 0.0508 | 14 | 101 |
| Deyang | 3.456 | 0.0413 | 12 | 83 |
| Mianyang | 4.868 | 0.0582 | 16 | 125 |
| Guangyuan | 2.306 | 0.0276 | 8 | 62 |
| Suining | 2.814 | 0.0336 | 9 | 77 |
| Neijiang | 3.141 | 0.0375 | 11 | 71 |
| Leshan | 3.16 | 0.0378 | 11 | 175 |
| Nanchong | 5.608 | 0.0670 | 19 | 141 |
| Meishan | 2.955 | 0.0353 | 10 | 75 |
| Yibin | 4.589 | 0.0548 | 15 | 270 |
| Guang'an | 3.255 | 0.0389 | 11 | 109 |
| Dazhou | 5.385 | 0.0644 | 18 | 141 |
| Ya'an | 1.435 | 0.0171 | 5 | 66 |
| Bazhong | 2.713 | 0.0324 | 9 | 71 |
| Ziyang | 2.309 | 0.0276 | 8 | 45 |
| Aba Tibetan and Qiang Autonomous Prefecture | 0.823 | 0.0098 | 3 | 19 |
| Ganzi Tibetan Autonomous Prefecture | 1.107 | 0.0132 | 4 | 30 |
| Liangshan | 4.858 | 0.0581 | 16 | 128 |
| Total | 83.675 | 1.0000 | 282 | 2529 |

*Note.* Data source of “Population” is from Sichuan Provincial Health Statistical Yearbook (2020). Stratified proportional sampling is used to calculate the minimum sample size of each city in Sichuan Province.

**Table S2** Area distribution of the minimum travel time to obtain LDCT services in different types of healthcare institutions

| Minimum Travel Time (min) | 0-15 | 15-30 | 30-60 | 60-120 | >120 |
| --- | --- | --- | --- | --- | --- |
| Driving/Public transportation | | | | | |
| Proportion of total coverage area | 13.18% | 18.57% | 24.28% | 23.69% | 20.28% |
| Proportion of healthcare institutions above provincial-level | 0.81% | 2.16% | 8.34% | 18.44% | 70.25% |
| Proportion of municipal-level healthcare institutions | 2.58% | 7.49% | 17.54% | 21.16% | 51.22% |
| Proportion of county-level healthcare institutions | 10.44% | 18.07% | 25.31% | 25.52% | 20.66% |
| Proportion of other healthcare institutions | 8.76% | 12.96% | 17.00% | 16.14% | 45.14% |
| Bicycle/Electric vehicle | | | | | |
| Proportion of total coverage area | 1.22% | 2.74% | 7.81% | 15.80% | 72.43% |
| Proportion of healthcare institutions above provincial-level | 0.06% | 0.17% | 0.55% | 1.47% | 97.75% |
| Proportion of municipal-level healthcare institutions | 0.17% | 0.44% | 1.48% | 5.07% | 92.83% |
| Proportion of county-level healthcare institutions | 0.67% | 1.88% | 6.29% | 14.98% | 76.16% |
| Proportion of other healthcare institutions | 0.77% | 1.84% | 5.40% | 11.30% | 80.70% |
| Walking | | | | | |
| Proportion of total coverage area | 0.18% | 0.44% | 1.39% | 3.97% | 94.02% |
| Proportion of healthcare institutions above provincial-level | 0.01% | 0.02% | 0.08% | 0.28% | 99.61% |
| Proportion of municipal-level healthcare institutions | 0.02% | 0.06% | 0.21% | 0.69% | 99.01% |
| Proportion of county-level healthcare institutions | 0.07% | 0.23% | 0.87% | 2.92% | 95.90% |
| Proportion of other healthcare institutions | 0.10% | 0.27% | 0.92% | 2.78% | 95.93% |

**Table S3** Urban-rural differences in the minimum travel time to obtain LDCT services

| Minimum Travel Time (min) | | 0-15 | 15-30 | 30-60 | 60-120 | >120 |
| --- | --- | --- | --- | --- | --- | --- |
| Proportion of high-risk population | | | | | | |
| Driving/Public transportation | Urban | 73.71% | 18.57% | 7.03% | 0.67% | 0.02% |
|  | Rural | 43.86% | 31.69% | 19.07% | 4.45% | 0.94% |
| Bicycle/Electric vehicle | Urban | 34.73% | 16.88% | 20.27% | 19.76% | 8.36% |
|  | Rural | 12.03% | 9.49% | 19.67% | 33.45% | 25.36% |
| Walking | Urban | 10.87% | 14.26% | 16.51% | 18.06% | 40.30% |
|  | Rural | 2.89% | 5.01% | 7.37% | 12.02% | 72.71% |
| Area proportion | | | | | | |
| Driving/Public transportation | Urban | 32.38% | 30.43% | 21.83% | 12.04% | 3.32% |
|  | Rural | 9.09% | 16.04% | 24.83% | 26.18% | 23.86% |
| Bicycle/Electric vehicle | Urban | 4.05% | 7.83% | 18.41% | 28.75% | 40.96% |
|  | Rural | 0.62% | 1.65% | 5.56% | 13.05% | 79.12% |
| Walking | Urban | 0.64% | 1.48% | 4.34% | 11.11% | 82.43% |
|  | Rural | 0.08% | 0.21% | 0.76% | 2.45% | 96.50% |

**Table S4** Population distribution of the minimum travel time to obtain LDCT services in different types of healthcare institutions

| Minimum Travel Time (min) | | 0-15 | 15-30 | 30-60 | 60-120 | >120 |
| --- | --- | --- | --- | --- | --- | --- |
| Driving/Public transportation | | | | | | |
| Proportion of total coverage area | Urban | 73.71 | 18.57 | 7.03 | 0.67 | 0.02 |
|  | Rural | 43.86 | 31.69 | 19.07 | 4.45 | 0.94 |
| Proportion of healthcare institutions above provincial-level | Urban | 26.70 | 14.85 | 26.04 | 24.08 | 8.33 |
|  | Rural | 0.67 | 4.13 | 28.63 | 44.21 | 22.35 |
| Proportion of municipal-level healthcare institutions | Urban | 43.41 | 31.63 | 20.89 | 3.82 | 0.25 |
|  | Rural | 2.87 | 18.58 | 49.51 | 23.16 | 5.88 |
| Proportion of county-level healthcare institutions | Urban | 64.27 | 24.80 | 10.04 | 0.87 | 0.03 |
|  | Rural | 35.58 | 33.03 | 24.43 | 5.95 | 1.00 |
| Proportion of other healthcare institutions | Urban | 66.51 | 21.32 | 11.05 | 0.99 | 0.14 |
|  | Rural | 31.48 | 30.62 | 26.83 | 8.06 | 3.00 |
| Bicycle/Electric vehicle | | | | | | |
| Proportion of total coverage area | Urban | 34.73 | 16.88 | 20.27 | 19.76 | 8.36 |
|  | Rural | 12.03 | 9.49 | 19.67 | 33.45 | 25.36 |
| Proportion of healthcare institutions above provincial-level | Urban | 6.69 | 6.84 | 10.94 | 12.61 | 62.93 |
|  | Rural | 0.08 | 0.07 | 0.48 | 2.14 | 97.22 |
| Proportion of municipal-level healthcare institutions | Urban | 13.73 | 11.53 | 14.29 | 25.84 | 34.61 |
|  | Rural | 0.35 | 0.59 | 1.12 | 8.43 | 89.50 |
| Proportion of county-level healthcare institutions | Urban | 17.49 | 18.84 | 25.57 | 25.04 | 13.06 |
|  | Rural | 8.95 | 7.81 | 15.21 | 32.31 | 35.72 |
| Proportion of other healthcare institutions | Urban | 27.59 | 16.69 | 19.51 | 21.01 | 15.21 |
|  | Rural | 7.68 | 6.97 | 14.83 | 28.06 | 42.46 |
| Walking | | | | | | |
| Proportion of total coverage area | Urban | 10.87 | 14.26 | 16.51 | 18.06 | 40.30 |
|  | Rural | 2.89 | 5.01 | 7.37 | 12.02 | 72.71 |
| Proportion of healthcare institutions above provincial-level | Urban | 1.40 | 2.60 | 5.11 | 8.64 | 82.25 |
|  | Rural | 0.00 | 0.04 | 0.06 | 0.21 | 99.69 |
| Proportion of municipal-level healthcare institutions | Urban | 2.57 | 5.61 | 10.22 | 11.50 | 70.09 |
|  | Rural | 0.03 | 0.13 | 0.39 | 0.79 | 98.66 |
| Proportion of county-level healthcare institutions | Urban | 2.78 | 7.11 | 14.32 | 21.78 | 54.00 |
|  | Rural | 1.70 | 3.68 | 6.35 | 9.41 | 78.86 |
| Proportion of other healthcare institutions | Urban | 7.35 | 11.53 | 15.16 | 17.99 | 47.97 |
|  | Rural | 1.80 | 3.08 | 5.21 | 9.04 | 80.87 |

**Table S5** Area distribution of the minimum travel time to obtain LDCT services in different types of healthcare institutions

| Minimum Travel Time (min) | | 0-15 | 15-30 | 30-60 | 60-120 | >120 |
| --- | --- | --- | --- | --- | --- | --- |
| Driving/Public transportation | | | | | | |
| Proportion of total coverage area | Urban | 32.38 | 30.43 | 21.83 | 12.04 | 3.32 |
|  | Rural | 9.09 | 16.04 | 24.83 | 26.18 | 23.86 |
| Proportion of healthcare institutions above provincial-level | Urban | 4.37 | 9.25 | 20.52 | 28.77 | 37.09 |
|  | Rural | 0.04 | 0.64 | 5.73 | 16.20 | 77.39 |
| Proportion of municipal-level healthcare institutions | Urban | 12.47 | 27.10 | 32.41 | 17.42 | 10.60 |
|  | Rural | 0.45 | 3.27 | 14.34 | 21.95 | 59.99 |
| Proportion of county-level healthcare institutions | Urban | 24.48 | 30.36 | 26.00 | 14.51 | 4.65 |
|  | Rural | 7.47 | 15.47 | 25.16 | 27.89 | 24.02 |
| Proportion of other healthcare institutions | Urban | 26.00 | 29.31 | 22.51 | 8.89 | 13.29 |
|  | Rural | 5.07 | 9.45 | 15.78 | 17.69 | 52.01 |
| Bicycle/Electric vehicle | | | | | | |
| Proportion of total coverage area | Urban | 4.05 | 7.83 | 18.41 | 28.75 | 40.96 |
|  | Rural | 0.62 | 1.65 | 5.56 | 13.05 | 79.12 |
| Proportion of healthcare institutions above provincial-level | Urban | 0.33 | 0.92 | 2.95 | 6.71 | 89.09 |
|  | Rural | 0.00 | 0.01 | 0.03 | 0.34 | 99.61 |
| Proportion of municipal-level healthcare institutions | Urban | 0.87 | 2.20 | 7.34 | 20.92 | 68.67 |
|  | Rural | 0.02 | 0.06 | 0.22 | 1.67 | 98.03 |
| Proportion of county-level healthcare institutions | Urban | 1.86 | 5.09 | 15.42 | 29.11 | 48.52 |
|  | Rural | 0.42 | 1.20 | 4.36 | 11.98 | 82.03 |
| Proportion of other healthcare institutions | Urban | 2.75 | 5.85 | 15.06 | 25.80 | 50.55 |
|  | Rural | 0.34 | 0.98 | 3.34 | 8.19 | 87.15 |
| Walking | | | | | | |
| Proportion of total coverage area | Urban | 0.64 | 1.48 | 4.34 | 11.11 | 82.43 |
|  | Rural | 0.08 | 0.21 | 0.76 | 2.45 | 96.50 |
| Proportion of healthcare institutions above provincial-level | Urban | 0.05 | 0.11 | 0.43 | 1.51 | 97.90 |
|  | Rural | 0.00 | 0.00 | 0.01 | 0.01 | 99.98 |
| Proportion of municipal-level healthcare institutions | Urban | 0.10 | 0.31 | 1.08 | 3.47 | 95.03 |
|  | Rural | 0.00 | 0.01 | 0.03 | 0.10 | 99.87 |
| Proportion of county-level healthcare institutions | Urban | 0.22 | 0.63 | 2.44 | 8.13 | 88.59 |
|  | Rural | 0.04 | 0.14 | 0.54 | 1.82 | 97.46 |
| Proportion of other healthcare institutions | Urban | 0.39 | 0.99 | 3.09 | 8.68 | 86.86 |
|  | Rural | 0.04 | 0.11 | 0.46 | 1.52 | 97.87 |

**Table S6** Minimum travel time to obtain LDCT services in 21 cities and prefectures by driving/public transportation

| Cities (Prefectures) | Population proportion | | | | | Area proportion | | | | |
| --- | --- | --- | --- | --- | --- | --- | --- | --- | --- | --- |
|  | 0-15min | 15-30min | 30-60min | 60-120min | >120min | 0-15min | 15-30min | 30-60min | 60-120min | >120min |
| Aba Tibetan and Qiang Autonomous Prefecture | 26.01 | 18.03 | 26.65 | 21.69 | 7.63 | 3.28 | 10.53 | 30.43 | 39.24 | 16.52 |
| Bazhong | 30.06 | 29.18 | 34.25 | 6.50 | 0.01 | 11.69 | 28.59 | 41.92 | 17.60 | 0.21 |
| Chengdu | 89.74 | 7.99 | 2.25 | 0.02 | 0.00 | 57.82 | 24.52 | 15.43 | 2.24 | 0.00 |
| Dazhou | 42.17 | 29.84 | 23.44 | 4.55 | 0.00 | 20.62 | 31.01 | 34.40 | 13.97 | 0.00 |
| Deyang | 76.88 | 20.69 | 2.40 | 0.03 | 0.00 | 50.96 | 32.65 | 10.90 | 5.49 | 0.00 |
| Ganzi Tibetan Autonomous Prefecture | 19.70 | 13.04 | 13.77 | 26.02 | 27.48 | 2.34 | 5.59 | 14.30 | 29.44 | 48.34 |
| Guang'an | 55.52 | 38.67 | 5.82 | 0.00 | 0.00 | 38.85 | 48.23 | 12.92 | 0.00 | 0.00 |
| Guangyuan | 35.21 | 25.14 | 35.77 | 3.89 | 0.00 | 13.57 | 30.13 | 45.72 | 10.58 | 0.00 |
| Leshan | 71.55 | 19.83 | 8.14 | 0.49 | 0.00 | 29.52 | 29.10 | 27.57 | 13.81 | 0.00 |
| Liangshan Yi Autonomous Prefectural | 33.11 | 19.74 | 24.21 | 19.84 | 3.10 | 7.16 | 14.80 | 29.63 | 29.75 | 18.66 |
| Luzhou | 53.27 | 25.47 | 17.25 | 4.01 | 0.00 | 25.34 | 29.40 | 34.21 | 11.05 | 0.00 |
| Meishan | 60.33 | 34.67 | 4.72 | 0.28 | 0.00 | 37.04 | 40.88 | 15.04 | 7.04 | 0.00 |
| Mianyang | 48.20 | 32.13 | 15.91 | 3.28 | 0.49 | 16.03 | 26.11 | 23.02 | 31.98 | 2.86 |
| Neijiang | 63.80 | 28.05 | 8.15 | 0.00 | 0.00 | 45.28 | 41.21 | 13.52 | 0.00 | 0.00 |
| Nanchong | 49.57 | 31.25 | 18.72 | 0.45 | 0.00 | 32.80 | 40.62 | 25.84 | 0.74 | 0.00 |
| Panzhihua | 78.38 | 14.94 | 4.88 | 1.80 | 0.00 | 18.65 | 34.50 | 33.54 | 13.31 | 0.00 |
| Suining | 51.98 | 39.35 | 8.67 | 0.00 | 0.00 | 38.89 | 50.30 | 10.82 | 0.00 | 0.00 |
| Ya'an | 64.76 | 25.51 | 8.88 | 0.86 | 0.00 | 17.13 | 30.90 | 39.45 | 12.52 | 0.00 |
| Yibin | 54.83 | 30.08 | 13.49 | 1.60 | 0.00 | 32.43 | 38.75 | 25.38 | 3.44 | 0.00 |
| Ziyang | 37.74 | 37.49 | 24.77 | 0.00 | 0.00 | 28.68 | 45.28 | 26.04 | 0.00 | 0.00 |
| Zigong | 64.47 | 31.08 | 4.45 | 0.00 | 0.00 | 43.68 | 47.45 | 8.87 | 0.00 | 0.00 |

**Table S7** Proportion of high-risk population covered by different types of healthcare institutions within a 60-minute driving/public transportation range

| Cities (Prefectures) | Proportion of healthcare institutions above provincial-level | Proportion of municipal-level healthcare institutions | Proportion of county-level healthcare institutions | Proportion of other healthcare institutions |
| --- | --- | --- | --- | --- |
| Aba Tibetan and Qiang Autonomous Prefecture | 7.56% | 5.89% | 65.52% | 21.10% |
| Bazhong | 0.00% | 69.32% | 90.69% | 92.12% |
| Chengdu | 99.03% | 99.70% | 99.97% | 99.98% |
| Dazhou | 2.77% | 76.39% | 93.95% | 93.57% |
| Deyang | 94.06% | 98.19% | 99.97% | 98.56% |
| Ganzi Tibetan Autonomous Prefecture | 0.00% | 15.82% | 46.34% | 0.01% |
| Guang'an | 59.39% | 97.67% | 100.00% | 100.00% |
| Guangyuan | 1.89% | 57.48% | 95.96% | 82.41% |
| Leshan | 0.13% | 80.62% | 99.50% | 93.34% |
| Liangshan Yi Autonomous Prefectural | 0.00% | 28.09% | 66.98% | 61.23% |
| Luzhou | 73.92% | 75.07% | 95.82% | 95.99% |
| Meishan | 62.76% | 99.40% | 99.68% | 99.71% |
| Mianyang | 81.66% | 84.28% | 96.23% | 94.50% |
| Neijiang | 34.13% | 99.91% | 100.00% | 100.00% |
| Nanchong | 65.81% | 79.29% | 99.00% | 95.60% |
| Panzhihua | 0.00% | 94.20% | 97.62% | 90.50% |
| Suining | 63.51% | 99.45% | 100.00% | 100.00% |
| Ya'an | 24.18% | 83.09% | 98.36% | 96.36% |
| Yibin | 19.76% | 69.82% | 96.04% | 98.34% |
| Ziyang | 17.13% | 86.84% | 99.68% | 100.00% |
| Zigong | 50.71% | 99.51% | 99.99% | 100.00% |
